# New insights into the molecular biology of Alzheimer’s-like cerebral amyloidosis achieved through multi‐omics approaches

**DOI:** 10.1371/journal.pone.0330859

**Published:** 2025-09-03

**Authors:** Lorenzo Campanelli, Juan M. Sendoya, Scott Brody, Pablo Galeano, Sonia Do Carmo, A. Claudio Cuello, Eduardo M. Castaño, Andrés Gonzalés-Jimenez, Julia Verheul, Dina Medina-Vera, Fernando Rodríguez de Fonseca, Rasmus Wernersson, Laura Morelli

**Affiliations:** 1 Laboratory of Brain Aging and Neurodegeneration, Fundación Instituto Leloir, Ciudad Autónoma de Buenos Aires, Argentina; 2 ZS Discovery department, ZS Associates, Buenos Aires, Argentina; 3 ZS Discovery department, ZS Associates, Illinois, Evanston, United States of America; 4 Department of Pharmacology and Therapeutics, McGill University, Montreal, Quebec, Canada; 5 Grupo de Neuropsicofarmacología, Instituto de Investigación Biomédica de Málaga y Plataforma en Nanomedicina (IBIMA-Plataforma BIONAND), Málaga, Spain; 6 Unidad de Gestión Clínica de Neurología, Hospital Regional Universitario de Málaga, Málaga, Spain; 7 ZS Discovery department, ZS Associates, Kongens Lyngby, Denmark; University of Jeddah, SAUDI ARABIA

## Abstract

**Background:**

One of the neuropathologic hallmarks of Alzheimer’s disease (AD) is amyloid plaques composed of fibrillar amyloid beta (Aβ) that accumulate in the hippocampus and cerebral cortex. The identification of molecular changes and interactions associated with Aβ-dependent cerebral amyloidosis is a need in the field. We hypothesize that structured datasets linking proteins to differentially abundant metabolites may provide an indirect but effective means of elucidating the processes and functions in which these metabolites are involved. The goal of this study was to identify core network modules related to AD-like cerebral amyloidosis to provide new insights into the molecular underpinnings of this brain disorder potentially associated with diet and microbiota modulation.

**Methods:**

We performed fecal bacterial genotyping and untargeted metabolomic analysis of plasma and feces from wild-type and McGill-R-Thy1-APP transgenic (Tg) rats, a model of AD-like cerebral amyloidosis, that were exposed to a high-fat diet protocol. To identify relevant proteins associated with the discriminant metabolites, we used several structured databases. Protein-metabolite associations (both physical and functional) were retrieved, and a collection of AD-associated protein-protein interaction (PPI) networks were built using a near-neighborhood approach.

**Results:**

A total of 44 bacterial genera and 636 plasma and 576 fecal metabolites were analyzed. From the discriminating metabolites of the Sparse Partial Least Squares Discriminant Analysis (sPLS-DA) models, 657 networks were collected and a subset of the top 20 exploratory networks was defined. The first ranked network in terms of seed protein enrichment and number of participating metabolites showed strong biological signals of innate and adaptive immunity processes, with CD36 emerging as a central hub, orchestrating immunity, metabolic pathways, and fatty acid trafficking.

**Conclusions:**

The network biology approach enabled a precise definition of the metabolic pathways underlying the disease biology highlighting the role of immune system in the complex interaction of the brain-gut axis.

## Introduction

Alzheimer’s disease (AD), the leading cause of dementia in the aged population, is a complex brain disorder with a significant impact on global health. Current estimates predict that dementia cases will double in Europe and triple worldwide by 2050 [1]. Moreover, 68% of the projected increase will take place in low- and middle-income countries, where it is more difficult to meet the overall cost of care [2]. The main pathophysiological features of AD are amyloid plaques, composed largely of fibrillar amyloid-β (Aβ), and neurofibrillary tangles, composed mainly of phosphorylated tau (p-tau) [3,4].

Recent research on AD has made significant progress in understanding its molecular and cellular mechanisms, particularly the role of Aβ and p-tau as well as glial dysfunction [5,6]. Although the precise molecular forms of accumulated Aβ in the hippocampus and cortex in AD brains remains unclear [7], the favorable results of recent treatments that target Aβ for patients in the early stages of AD pathology [8] emphasize the necessity to identify molecular alterations and interactions linked to Aβ-dependent cerebral amyloidosis.

Latest studies suggests that the gut microbiota plays a significant role in the onset and progression of AD. It is becoming increasingly clear from recent studies that gut dysbiosis, or alterations in the microbiome, may contribute to the development of AD through several mechanisms. These include neuroinflammation, immune dysregulation, abnormal protein aggregation and impaired barrier permeability in both the intestine and brain [9]. The gut-brain axis, a bidirectional communication system, is implicated in this process, with microbiota metabolites potentially affecting cognitive decline associated with AD [10]. However, current research is constrained by a lack of understanding of the intricate interplay between microbiota metabolites and cerebral amyloid pathology.

The use of multi-omics approaches has the potential to uncover molecular alterations and interactions underlying complex diseases [11]. The “omics era” has revolutionized the study of AD, providing the opportunity to investigate the relationship between molecular pathways and to discover novel diagnostic biomarkers or targets for the development of specific drugs [12]. Results from genome-wide association studies (GWAS) have identified more than 80 loci whose variants have been reported to be associated with AD [13] and several processes involved in the development of the disease, including TNF signaling and activation of leukocytes, complement and microglia [14–16].

In addition, metabolomics has been used to identify potential biomarkers and pathways associated with AD onset and progression [17,18]. Perturbations in key pathways, including energy metabolism, the urea cycle and neurotransmission, have been identified [19]. Studies have shown that metabolomic changes in both blood and brain are associated with AD, with some metabolites potentially serving as biomarkers of the disease [20]. On the other hand, the fecal metabolome provides a functional readout of gut microbial activity and is strongly associated with visceral fat mass, providing insight into microbiome-associated phenotypes [21]. Microbial metabolites play a critical role in human health and represent potential sources of new therapeutic agents [22]. However, metaproteomic studies face challenges due to the complexity and diversity of microbiome samples [23].

Although several statistical tools and methods have been developed to identify pathways linked to a group of metabolites [24,25], post hoc analysis and pathway analysis are exclusively linked to the decision of the researcher based on the publicly available database. It should be noted that one of the main limitations of metabolomic studies is the scarcity of metabolite pathway information in high quality databases.

Pathway information from sources such as the Kyoto Encyclopedia of Genes and Genomes (KEGG), WikiPathways and Reactome is often of a high quality and is a valuable resource for interpreting biological data. It is important to note that these pathways often represent well-established, “textbook-level” knowledge. They are curated summaries based on scientific consensus, so it is unlikely that novel interactions between genes/proteins associated with AD-like cerebral amyloidosis will be discovered by relying solely on pathway information.

We hypothesize that metabolite-to-protein data can serve as a proxy to overcome the scarcity of highly curated metabolite-to-biological process annotations. Specifically, the use of structured data linking proteins to differentially abundant metabolites may provide an indirect yet effective means to elucidate the processes and functions in which these metabolites are involved. This approach aims to bridge the gap in metabolite-to-pathway mapping by using protein associations as a functional intermediary. Since proteins do not act independently, the study of protein-protein interaction (PPI) networks can provide valuable insights into disease biology. PPI networks cover a broader spectrum of human proteins than traditional pathway databases, reaching up to 85% proteome coverage [26,27]. PPI are especially adept at revealing connections outside of established pathways because they stem from high-throughput experimental data rather than literature curation. Through network analysis of PPI data, “proto-pathways” or biological modules may emerge that can eventually be validated and evolve into canonical pathways.

However, when working with PPI data, it is crucial to consider potential experimental artifacts that may affect the results. In this study, we focused on two important aspects: 1) the high false-positive rate in PPI data makes it essential to use high-confidence resources where the evidence for each interaction has been evaluated [28]; and 2) over-connected proteins, which may appear to interact with many partners due to bias or experimental error, can skew the analysis. These highly connected proteins often show up in studies of different diseases, such as AD, heart disease, or cancer, but may not be relevant to the specific disease being studied.

The overall goal of this work was to identify core network modules related to AD-like cerebral amyloidosis to provide both confirmation of known biology and new insights into the molecular underpinnings of this brain disorder potentially associated with diet and microbiota modulation [29]. Our experimental approach allowed us to identify 20 core network modules associated with AD-like cerebral amyloidosis and dietary modulation, that support known biological processes and provide new insights into the underlying mechanisms of cerebral amyloidosis.

## Materials and methods

### 1. Data collection

#### 1.1. Animals and ethical implications.

McGill transgenic (Tg) rats, a model of AD-like cerebral amyloidosis, were provided to the Leloir Institute Foundation (FIL) by The Royal Institution for the Advancement of Learning/McGill University, Canada, through the signing of an MTA between FIL (Argentina) and McGill University (Canada). Tg rats express human APP751with the Swedish and Indiana mutations, under the control of the murine Thy1.2 promoter [30]. Homozygous Tg rats show intraneuronal Aβ accumulation until 6−9 months of age, when Aβ plaques appear in the hippocampus and cortex. This intra- and extracellular Aβ accumulation and deposition occurs in the absence of intraneuronal accumulation of hyperphosphorylated tau and neurodegeneration and is accompanied by cognitive deficits, making it an excellent model to study AD-like cerebral amyloidosis. Additionally, quantitative proteomics in this rat model revealed distinct changes in hippocampal protein expression at early and late stages of amyloid pathology, suggesting that different molecular pathways are affected throughout disease progression [31]. All experimental procedures were carried out in agreement with the guidelines of ARRIVE and OLAW-NIH. The protocol was approved by the local animal care committee (CICUAL # A5168-01).

#### 1.2. Nutritional paradigm and disease association.

The samples (plasma and stool) analyzed in this report were isolated from rats exposed to either a control diet (CD) or high-fat diet (HFD) over a period of six months. It was suggested that omega-6 is primarily proinflammatory, while omega-3 has anti-inflammatory effects in AD neuropathology [32]. Therefore, we addressed whether the intake of an HFD with an elevated omega-6-to-omega-3 ratio as compared to the CD (25:1 vs. 8.4:1) negatively impacts the behavioral and neuropathological characteristics of AD. Our results showed that HFD impaired reference memory in wild-type (WT) animals but did not worsen it in Tg. Furthermore, we found that the HFD did not cause obesity, and did not increase triglycerides or glucose levels. In contrast, the HDF promoted stronger microglial activation in Tg rats compared to WT rats, but had no effect on cerebral amyloid deposition [29].

#### 1.3. Experimental design.

**Fig 1:**
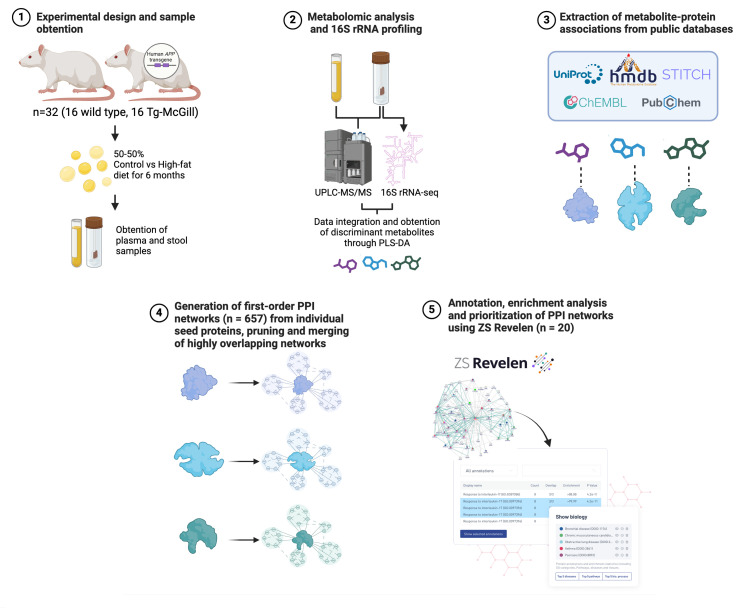
Experimental design and study workflow. Outline the workflow followed in this study. Briefly, plasma and stool samples of wild type and Tg-McGill animals were collected, and metabolomic analysis and 16sRNA profiling were performed to obtain discriminant metabolites through sPLS-DA. Metabolite-protein associations were extracted from public databases. First-order protein-protein interactions (PPI) were generated and networks with a large overlap were merged, producing a final set of 657 modules. “Core modules” (n = 20) were defined through over-representation analysis. Finally, analysis of distinct biological signals was performed to determine the most relevant ranked networks.

The rationale behind employing different diets was to elucidate the molecular pathways involved in the negative impact of HFD on behavior and neuropathology characteristics of AD-like cerebral amyloidosis. A schematic representation of the experimental design and study workflow is depicted in Fig 1. This includes the isolation of plasma and stool samples from WT (n = 16) and Tg (n = 16) rats exposed to CD or HFD followed by metabolic analysis and 16S rRNA profiling. Metabolite-protein associations were extracted from public databases and first-order protein-protein interaction (PPI) networks were generated from individual seed proteins. These networks were then pruned, and merged when they had high overlap. PPI network enrichment analysis and prioritization was performed using ZS Revelen, and distinct biological signals were analyzed. This workflow analysis provides a valuable framework for advancing precision medicine, offering insights into the mechanisms underlying AD-like cerebral amyloidosis through the integration of network biology strategies in multi-omics analyses.

#### 1.4. Metabolites data acquisition.

Frozen plasma (n = 23) and stool samples (n = 30) were sent to Metabolon (USA) to detect and quantify metabolites by Ultrahigh Performance Liquid Chromatography-Tandem Mass Spectroscopy (UPLC-MS/MS). Data extraction and peak-identification software, data processing tools for QC and compound identification were carried out by the company.

#### 1.5. Microbiota data acquisition.

Stool samples were collected and flash-frozen to preserve microbial DNA. These samples were sent to StarSEQ GmbH (Germany) for 16S microbial rRNA profiling, targeting the V3-V4 region, using Illumina sequencing. Quality filtering was conducted using the DADA2 plugin implemented in QIIME2 (version 2023.5.0) [33,34]. The target amplicon region (V3–V4 of the 16S rRNA gene, between primers 341F and 806R) has an expected length of approximately 460 bp. With the applied truncation lengths (260 bp forward + 215 bp reverse), the expected overlap between reads is 15 bp. DADA2 was further employed to remove low-quality sequences and identify unique amplicon sequence variants (ASVs). A Phred score threshold of 25 was used as a quality cutoff, corresponding to ~99.7% base call accuracy. Detailed information on raw sequence counts, read filtering thresholds, and the number of reads retained at each step of the pipeline is provided in Supplementary Materials (S1 Appendix). Taxonomic classification was performed using the SILVA 138.2 database and the RESCRIPt tool (S1 Appendix) for the V3-V4 region [35,36]. The default confidence threshold of 0.7 was used, meaning that only classifications with ≥70% confidence were retained. All ASVs were successfully classified at least to the phylum level; no reads were labeled as “Unassigned”. For features not resolved at deeper taxonomic levels (e.g., family or genus), classification was retained at the highest informative level. Finally, the generated taxonomy table and associated plots were exported to RStudio for statistical analysis.

### 2. Procedure for obtaining discriminant analytes highly correlated with bacterial genera and diets

#### 2.1. Quality control and normalization of the analytes.

A total of 685 plasma metabolites and 702 fecal metabolites were processed (S1 Table). Metabolites with missing information greater than 30% were eliminated. To normalize the data, we followed the procedures used by Metabolon (USA) as follows, data were rescaled to a median of one, and plasma data were adjusted by sample volume. Missing values were imputed using half the minimum value across all batches in the median-scaled data. Finally, natural log-transformation was applied to the normalized and imputed data for subsequent statistical analyses. After processing, the data were captured in a PCA for outlier detection.

#### 2.2. Microbiome data processing.

A total of 120 bacterial genera (S2 Table), comprising 249,493 ASV counts were processed. Microbiota ASVs data was pre-processed as suggested in mixOmics (https://mixomics.org/mixmc/mixmc-preprocessing/). ASVs with counts less than 10% and median = 0 were filtered out. The ASVs counts were transformed to Centered Log Ratio (CLR). After processing, the data were captured in a PCA for outlier detection.

#### 2.3. Sparse Partial Least Squares Discriminant Analysis (sPLS-DA) data integration.

Stool microbiota, and stool and plasma metabolome data were integrated and analyzed using Data Integration Analysis for Biomarker discovery using Latent cOmponents (DIABLO; mixOmics package), a supervised multivariate method that integrates multiple omics datasets to identify correlated features across data types while discriminating between predefined groups, following the mixOmics pipeline [37]. For this analysis, it is required to have the 3 complete omics data sets for each animal. Based on the amount of available material analyzed, 22 animals were used for further analysis. After pre-processing de metabolomic data, one outlier was identified and removed after exceeding the 99th percentile of a chi-squared distribution (df = 2) using the squared Mahalanobis distance with two principal components (S1 Fig). As a result, twenty-one rats (6 WT exposed to CD, 3 WT exposed to HFD, 6 Tg exposed to CD and 6 Tg exposed to HFD) were used for integration analysis. Tuning was performed to select variables for the sparse model. Artificial test sets were created using cross-validation to optimize model parameters. The model was trained on multiple subsets (fold = 3) and evaluated on the remaining subset, iterating this process until each subset was used for evaluation (n repeat = 10). The optimal parameters were applied to the entire training set, and the model’s performance was assessed numerically and visually. Permutation test was performed on the entire dataset to assess overfitting. Plots were done with mixOmics package [38]. By default, Euclidean distance and Complete linkage methods were used for heatmap plot. Multiple sPLS-DA were performed to obtain discriminant variables. Discriminant metabolites obtained were used for origin analysis in MetOrigin (https://metorigin.met-bioinformatics.cn/home/) and for further association with relevant proteins.

### 3. PPI network analysis in the context of AD-like cerebral amyloidosis

#### 3.1. Extraction of metabolite-protein associations from public data sources.

To identify relevant proteins associated with the discriminant metabolites detected in this study, we utilized several structured databases, including UniProt (for protein-small molecule interaction data), PubChem (Bioassay data), Human Metabolome Database (HMDB), ChEMBL, and (Search Tool for Interacting Chemicals) STITCH [39–43]. All databases were accessed in June 2024 to ensure data consistency and reproducibility. For comprehensive metabolite-to-protein mapping, multiple metabolite identifiers were queried, including chemical names, synonyms, InChIKeys, Simplified Molecular Input Line Entry System (SMILES), and database-specific identifiers. Protein associations were classified as either physical interactions (e.g., direct binding) or functional associations (e.g., involvement in shared biological processes). Insufficiently supported associations, such as unclear text-mining co-mentions, were excluded to maintain data reliability and accuracy. The seed protein list was intentionally designed to be inclusive and comprehensive, as further filtering criteria were applied during network generation.

#### 3.2. Generation of AD-associated PPI networks.

For the network resource we utilized the inBio Map interactome [27] (now part of ZS Revelen - https://revelen.zsservices.com/). The August 2023 version of the interactome was used, with interactions filtered to include only those meeting a confidence score threshold of 0.1. The AD network collection was generated using the bottom-up approach described by Jensen et al. [44]. Briefly, the list of proteins associated to the metabolites from the previous step, was used as “seed proteins” to extract all first order networks around each one of the seed proteins individually. Networks were then pruned to remove over-connected nodes (proteins with a disproportionately high number of interactions outside the current network). Finally, networks with substantial overlap (>75%) were merged. Most networks were capped at a maximum of 200 nodes; however, seven larger networks (S2 Appendix) were retained due to their strong enrichment scores, which preserved relevant biological signals. This process resulted in networks that contained one or more seed proteins and had low inter-network overlap with the exception of two networks that we decided to keep because they originated from distinct groups based on variation patterns, diet or genotype (S3 Appendix). This workflow mitigates the influence of promiscuous interactions, ensuring they do not drive the identification of the AD modules. Our network generation process resulted in a collection of 657 networks, provided in. xgmml format as part of the supplementary materials (S4 Appendix).

### 4. Prioritization of key network modules

From the full set of 657 networks, we performed multiple over-representation analyses to evaluate the enrichment of specific gene sets. The gene sets used for these analyses, detailed in the supplementary materials (S5 Appendix), included AD-related gene sets from MSigDB, a comprehensive set of all seed proteins, and AD-related proteins extracted from the ZS Revelen network analysis tool (https://revelen.zsservices.com/). The over-representation analyses were conducted using one-sided Fisher’s exact tests to assess the enrichment of each gene set within the networks. To account for multiple testing, *p*-values were corrected using the Benjamini-Hochberg procedure. This analysis resulted in the identification of two main subsets of networks based on their enrichment profiles: Set #1- *AD Core Networks* (significantly enriched in AD-associated proteins) and Set # 2- *Exploratory Networks* (enriched in seed proteins). Since our goal was to find novel protein networks where differential metabolites converge in the experimental groups, all subsequent analyses were conducted on *Set # 2-Exploratory Networks.*

### 5. Biological annotation and visualization of the networks

The biological context of each network module in Set #2 was investigated using ZS Revelen, an online tool for exploring and visualizing protein-protein interactions and associated biological functions. Briefly, ZS Revelen automates enrichment analysis of PPI networks against public data resources including Gene Ontology, Reactome and WikiPathways as well as proprietary gene-to-disease text mining data sets and custom user definable gene sets. Using this resource, significant biological annotations for each network were identified and visualized.

## Results and discussion

### Discriminant analytes highly correlated with bacterial genera and diet

After data pre-processing, we worked with 636 plasma metabolites, 576 stool metabolites and 43 bacterial genera comprising 235,638 ASV counts. The selection of two components was based on an evaluation of the performance of sPLS-DA models with up to five components. sPLS-DA performs very well with two components; adding more does not improve the model any further (S2 Fig). As a first step, a tuning of multiple PLS-DA was evaluated. Due to the limited sample size of the WT-HF group (n = 3) and a computational limitation to process multiple models, the tuning algorithm did not converge in one *best model*. Therefore, we performed several sPLS-DA including sequentially different amounts of imputation data for the 636 plasma metabolites and 576 fecal metabolites with or without the 44 bacterial genera and we selected the discriminant metabolites of these models.

**Fig 2:**
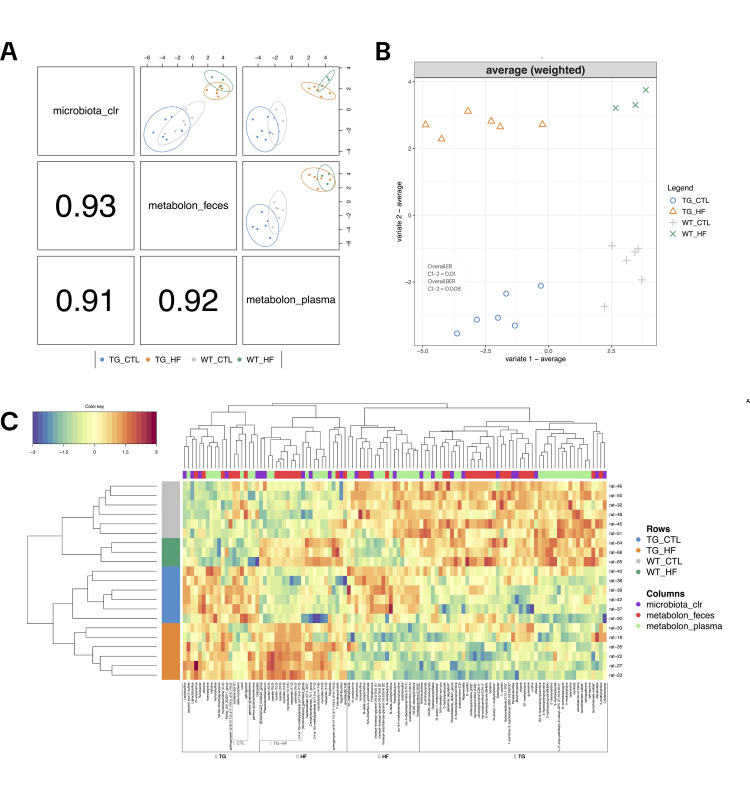
Discriminant metabolites in each experimental group. (A) Diagnostic plot from multiblock sPLS-DA. Samples are represented based on the specified component (here ncomp = 2) for each dataset (microbiota_clr, metabolon_feces and metabolon_plasma) and colored by experimental group. The 95% confidence ellipse plots are represented. The bottom left numbers indicate the correlation coeﬃcients between the first components from each dataset. (B) Sample plot from sPLS-DA. Samples are projected into the space spanned by the first two components showing discrimination between the experimental groups. Numbers in box represent the Classification Error Rate (Overall.ER) and Barret Error Rate (Overall.BER) scores. (C) Clustered Image Map (CIM) for the variables selected by best multiblock sPLS-DA performed on the study on component 1 and 2. The CIM represents samples in rows (indicated by their experimental group on the left side of the plot) and selected features in columns (indicated by their data type at the top of the plot).

From these models we obtained 79 metabolites (S3 Table) that were dysregulated between experimental groups. The final sPLS-DA model resulted in one that included 50 plasma metabolites, 48 fecal metabolites and 25 bacterial genera (S4 Table) with a high correlation between them (Fig 2A). The literature suggests that a proportion of the circulating metabolome in the body is derived from the microbiota [45]. For this reason, in this report we added the microbiota 16S dataset to sPLS-DA. By removing metabolites with the same ID in both feces and plasma (n = 7) from the final model, a total of 72 discriminative metabolites were included in the metOrigin analysis—a web tool that traces the potential sources of metabolites using multiple databases (HMDB, KEGG, FoodDB, DrugBank, T3DB, ChEBI, and BIGG). Of these, 40 metabolites were successfully traced (S5 Table) and share more than one category of association, while 32 could not be traced due to either a lack of information in the databases or the absence of an associated HMDB or KEGG ID. Among the 40 traced metabolites, 17 were associated with co-host-microbiota origin, and 6 were exclusively linked to microbiota. Additionally, 40 metabolites were related to food sources, and 4 were associated with environmental origins (S3 Fig). The final sPLS-DA model had a Barret Classification Rate (BER) < 0.01 using components 1 and 2, resulting in a remarkable classification of the 4 experimental groups (Fig 2B). To further analyze the experimental data, we performed a Clustered Image Map (CIM) to represent the expression of the multi-omic signature of each sample. As shown in Fig 2C, the areas of homogeneous expression levels of the animals across the metabolomic and microbial dataset can be determined. We performed a permutation test with 100,000 permutations, evaluating performance with the parameters set to folds = 3 and nrepeat = 10 and including all variables. The test yielded a p-value < 0.0001, indicating that the model is not overfitting the data.

Studying the range of the 72 metabolites (S5 Table) we found that 4 analytes including histidine, 2-aminobutyrate, cysteine and 3-hydroxybutyrate (BHBA) were linked with AD according to the databases used by Enrichment Analysis of Metaboanalyst 6.0 (https://www.metaboanalyst.ca/) in the Disease signatures option. We detected increases in histidine in Tg, in contrast to some studies that found decreased levels of this amino acid in AD patients compared to controls [46,47], but in agreement with other research that reported increased levels of 3-methylhistidine in AD samples [48]. These findings suggest a potential association between altered amino acid metabolism and the cognitive decline observed in AD, though further mechanistic and experimental validation is needed to establish causality. In addition, we found decreased levels of 2-aminobutyrate, cysteine and 3-hydroxybutyrate (BHB) in Tg as compared to WT. 2-aminobutyrate is a by-product of cysteine biosynthesis that modulates glutathione homeostasis by increasing intracellular glutathione levels and protecting against oxidative stress [49]. Reduced levels of 2-aminobutyrate may be associated with the generation of reactive oxygen species (ROS). Recently, the gaseous physiological modulator hydrogen sulfide (H2S) was shown to exert a variety of neuroprotective effects, and its plasma levels were reported to be markedly lower in AD patients than in matched controls. It is of note that the main enzyme that synthesizes H2S in the brain parenchyma, cystathionine beta-synthase employs cysteine as its rate-limiting substrate [50]. Therefore, decreased cysteine levels may be associated with impaired neuroprotective pathways. Recent reports highlight the role of ketone bodies (acetone, acetoacetate and BHB) in microglial metabolism and AD pathology, as they serve as an alternative energy source when glucose utilization is compromised. Moreover, increased BHB levels have been demonstrated to inhibit NLRP3 inflammasome activation, a key pathway in AD pathogenesis [51]. Additionally, it should be noted that the discriminating metabolites are primarily related to amino acids, lipids, vitamins, peptides, fatty acids, and energy metabolism. This is consistent with previous reports that have linked alterations in histidine, glutathione, cysteine, and ketone bodies with AD [52–54].

### Metabolite-Protein virtual pulldown and network collection

To explore the dynamic interactions between proteins and metabolites, we processed the differential metabolites obtained from the previous analysis (S3 Table) using publicly available structured data sources (UniProt, PubChem, HMDB, ChEMBL and STITCH). We identified metabolite-protein associations for 43 (S6 Table) out of 79 of the differentially abundant metabolites identified through sPLS-DAs. These associations involved 494 unique human proteins. Given the many-to-many nature of these associations, the total number of metabolite-protein interactions was 1,170 (S7 Table). These proteins were the seed proteins used for “virtual pulldowns”, which generated a final number of 657 protein networks (S4 Appendix). The concept “virtual pulldown” refers to the in-silico expansion of one or more seed proteins into a context-specific protein interaction subnetwork using curated interaction data. Specifically, it simulates the conceptual goal of a physical pulldown experiment (i.e., capturing functionally or physically associated proteins) by computationally extracting first-order interaction partners from a high-confidence interactome. From the filters mentioned previously (Prioritization of key network modules; Materials and Methods), we obtained 25 Core Networks from Set # 1 (AD core networks, Table 1), which comprises networks significantly enriched in AD-associated proteins (Benjamini-Hochberg corrected p-value, or q-value ≤ 0.05), and 20 Exploratory Networks from Set # 2 (Exploratory networks, Table 2), which includes networks enriched in seed proteins that were associated with three or more of the differentially abundant metabolites identified in the study. In both network sets, most proteins are primarily associated with differential metabolites in diet groups. Within these diet-associated networks, we also observed proteins linked to metabolites that varied in connection with the model genotype. This highlights the importance of diet in the context of disease. The exploratory networks exhibited significant biological signals reflecting processes occurring in the AD-like animal model in response to dietary intervention. In this study, we present two complementary approaches for exploring protein network tables. One focuses on filtering networks based on a trait of interest (Set #1), while the other aims to identify protein networks potentially affected in the model (Set #2). We selected the second approach (Set #2), as it emphasizes networks involving multiple metabolites, which are more likely to reflect novel biologically relevant changes in an animal model of AD-like amyloid pathology.

**Table 1:** AD-core networks.

Network Name	Size	Metabolites linked	Metabolites names	Q-Value
*c1_3_109*	36	2	linoleoyl-arachidonoyl-glycerol (18:2/20:4) [2]*;linoleoyl-linoleoyl-glycerol (18:2/18:2) [1]*	1.82E-08
*c1_3_116*	66	3	histidine;linoleoyl-arachidonoyl-glycerol (18:2/20:4) [2]*;linoleoyl-linoleoyl-glycerol (18:2/18:2) [1]*	0.000
*c1_3_42*	48	2	linoleoyl-arachidonoyl-glycerol (18:2/20:4) [2]*;linoleoyl-linoleoyl-glycerol (18:2/18:2) [1]*	3.77E-07
*c1_3_67*	82	6	cysteine;linoleoyl-arachidonoyl-glycerol (18:2/20:4) [2]*;linoleoyl-linoleoyl-glycerol (18:2/18:2) [1]*;margarate (17:0);myristate (14:0);palmitate (16:0)	3.77E-07
*c1_3_113*	50	2	linoleoyl-arachidonoyl-glycerol (18:2/20:4) [2]*;linoleoyl-linoleoyl-glycerol (18:2/18:2) [1]*	3.58E-06
*c1_3_115*	80	2	linoleoyl-arachidonoyl-glycerol (18:2/20:4) [2]*;linoleoyl-linoleoyl-glycerol (18:2/18:2) [1]*	4.97E-05
*c2_4_464*	38	5	linoleoyl-arachidonoyl-glycerol (18:2/20:4) [2]*;linoleoyl-linoleoyl-glycerol (18:2/18:2) [1]*;margarate (17:0);myristate (14:0);palmitate (16:0)	1.83E-04
*c1_3_107*	13	2	linoleoyl-arachidonoyl-glycerol (18:2/20:4) [2]*;linoleoyl-linoleoyl-glycerol (18:2/18:2) [1]*	3.57E-04
*c1_3_70*	193	5	linoleoyl-arachidonoyl-glycerol (18:2/20:4) [2]*;linoleoyl-linoleoyl-glycerol (18:2/18:2) [1]*;palmitate (16:0); 2-aminobutyrate; 1-(1-enyl-palmitoyl)-2-oleoyl-GPC (P-16:0/18:1)*	1.26E-03
*c1_3_106*	11	2	linoleoyl-arachidonoyl-glycerol (18:2/20:4) [2]*;linoleoyl-linoleoyl-glycerol (18:2/18:2) [1]*	2.40E-03
*c1_3_173*	16	2	linoleoyl-arachidonoyl-glycerol (18:2/20:4) [2]*;linoleoyl-linoleoyl-glycerol (18:2/18:2) [1]*	1.01E-02
*c2_4_457*	112	3	caprate (10:0);linoleoyl-arachidonoyl-glycerol (18:2/20:4) [2]*;linoleoyl-linoleoyl-glycerol (18:2/18:2) [1]*	1.01E-02
*c1_3_248*	221	1	trimethylamine N-oxide	8.61E-02
*c1_3_157*	10	2	linoleoyl-arachidonoyl-glycerol (18:2/20:4) [2]*;linoleoyl-linoleoyl-glycerol (18:2/18:2) [1]*	1.20E-01
*c1_3_246*	884	1	trimethylamine N-oxide	1.73E-01
*c2_4_406*	56	3	myristate (14:0);palmitate (16:0);laurate (12:0)	2.15E-01
*c2_4_586*	45	1	pantothenate	2.60E-01
*c2_4_590*	69	1	2-aminobutyrate	2.60E-01
*c2_4_415*	20	2	linoleoyl-arachidonoyl-glycerol (18:2/20:4) [2]*;linoleoyl-linoleoyl-glycerol (18:2/18:2) [1]*	4.34E-01
*c2_4_456*	603	11	2’-deoxycytidine;“acetylcarnitine (C2);laurate (12:0);sphingomyelin (d18:1/17:0, d17:1/18:0, d19:1/16:0)”;caprate (10:0);histidine;homoarginine;linoleoyl-arachidonoyl-glycerol (18:2/20:4) [2]*;linoleoyl-linoleoyl-glycerol (18:2/18:2) [1]*;margarate (17:0);palmitate (16:0)	4.70E-01
*c1_3_211*	23	1	palmitate (16:0)	4.76E-01
*c2_4_474*	334	7	2’-deoxycytidine;caprate (10:0);laurate (12:0);histidine;linoleoyl-arachidonoyl-glycerol (18:2/20:4) [2]*;linoleoyl-linoleoyl-glycerol (18:2/18:2) [1]*;palmitate (16:0)	5.55E-01
*c2_4_591*	603	1	laurate (12:0)	6.02E-01
*c1_3_249*	617	1	trimethylamine N-oxide	6.49E-01
*c2_4_623*	863	1	lysine	1.00E + 00

Network Name, ID of network. Size, number of proteins in the network. Metabolites linked, metabolites associated with network proteins. Q-value (Enrichment in seed protein list), enrichment value of metabolite-associated proteins over total proteins in the network. Cluster, Association cluster from the sPLS-DA. Diet, metabolites differentially expressed between CTL and HF diet; Genotype, metabolites differentially expressed between Tg and WT genotype; Mix, metabolites expressed in both diet and genotype. *, indicates ambiguity or incomplete structural characterization

**Table 2:** Exploratory networks.

*Network Name*	*Size*	*Metabolites linked*	*Metabolites names*	*Q-value*
*c1_3_65*	14	7	myristate (14:0);palmitate (16:0);linoleoyl-arachidonoyl-glycerol (18:2/20:4) [2]*;linoleoyl-linoleoyl-glycerol (18:2/18:2) [1]*;margarate (17:0);sphingomyelin (d18:1/17:0, d17:1/18:0, d19:1/16:0);laurate (12:0)	4.20E-04
*c1_3_14*	105	7	(14 or 15)-methylpalmitate (a17:0 or i17:0);caprate (10:0);laurate (12:0);myristoylcarnitine (C14);sphingomyelin (d18:1/17:0, d17:1/18:0, d19:1/16:0);myristate (14:0);palmitate (16:0)	1.72E-02
*c2_4_461*	60	7	3-hydroxybutyrate (BHBA);caprate (10:0);histidine;myristate (14:0);palmitate (16:0);laurate (12:0);margarate (17:0)	2.29E-05
*c1_3_67*	82	6	cysteine;linoleoyl-arachidonoyl-glycerol (18:2/20:4) [2]*;linoleoyl-linoleoyl-glycerol (18:2/18:2) [1]*;margarate (17:0);myristate (14:0);palmitate (16:0)	3.77E-07
*c1_3_43*	57	6	caprate (10:0);histidine;myristate (14:0);palmitate (16:0);laurate (12:0);margarate (17:0)	7.99E-05
*c1_3_63*	5	6	caprate (10:0);linoleoyl-arachidonoyl-glycerol (18:2/20:4) [2]*;linoleoyl-linoleoyl-glycerol (18:2/18:2) [1]*;margarate (17:0);myristate (14:0);palmitate (16:0)	3.87E-02
*c2_4_552*	8	5	2’-deoxycytidine;uridine;margarate (17:0);myristate (14:0);palmitate (16:0)	2.96E-05
*c2_4_464*	38	5	linoleoyl-arachidonoyl-glycerol (18:2/20:4) [2]*;linoleoyl-linoleoyl-glycerol (18:2/18:2) [1]*;margarate (17:0);myristate (14:0);palmitate (16:0)	1.83E-04
*c2_4_463*	9	5	myristate (14:0);palmitate (16:0);linoleoyl-arachidonoyl-glycerol (18:2/20:4) [2]*;linoleoyl-linoleoyl-glycerol (18:2/18:2) [1]*;margarate (17:0)	1.30E-02
*c2_4_418*	10	5	linoleoyl-arachidonoyl-glycerol (18:2/20:4) [2]*;linoleoyl-linoleoyl-glycerol (18:2/18:2) [1]*;margarate (17:0);myristate (14:0);palmitate (16:0)	1.71E-02
*c1_3_71*	95	5	caprate (10:0);margarate (17:0);laurate (12:0);myristate (14:0);palmitate (16:0)	2.65E-02
*c1_3_91*	13	5	caprate (10:0);laurate (12:0);myristate (14:0);palmitate (16:0);margarate (17:0)	2.66E-02
*c1_3_117*	29	5	caprate (10:0);margarate (17:0);laurate (12:0);myristate (14:0);palmitate (16:0)	3.87E-02
*c1_3_70*	193	5	linoleoyl-arachidonoyl-glycerol (18:2/20:4) [2]*;linoleoyl-linoleoyl-glycerol (18:2/18:2) [1]*;palmitate (16:0); 2-aminobutyrate; 1-(1-enyl-palmitoyl)-2-oleoyl-GPC (P-16:0/18:1)*	1.26E-03
*c1_3_62*	6	4	histidine;laurate (12:0);linoleoyl-arachidonoyl-glycerol (18:2/20:4) [2]*;linoleoyl-linoleoyl-glycerol (18:2/18:2) [1]*	1.90E-04
*c1_3_112*	24	4	caprate (10:0);laurate (12:0);myristate (14:0);palmitate (16:0)	2.36E-02
*c1_3_111*	5	4	adenine;myristate (14:0);margarate (17:0);palmitate (16:0)	3.87E-02
*c1_3_116*	66	3	histidine;linoleoyl-arachidonoyl-glycerol (18:2/20:4) [2]*;linoleoyl-linoleoyl-glycerol (18:2/18:2) [1]*	2.59E-07
*c2_4_457*	112	3	caprate (10:0);linoleoyl-arachidonoyl-glycerol (18:2/20:4) [2]*;linoleoyl-linoleoyl-glycerol (18:2/18:2) [1]*	1.01E-02
*c1_3_80*	19	3	3-hydroxybutyrate (BHBA);caprate (10:0);laurate (12:0)	1.30E-02

Network Name, ID of network. Size, number of proteins in the network. Metabolites linked, metabolites associated with network proteins. Q-value (Enrichment in seed protein list), enrichment value of metabolite-associated proteins over total proteins in the network. Cluster, Association cluster from the sPLS-DA. Diet, metabolites differentially expressed between CTL and HF diet; Genotype, metabolites differentially expressed between Tg and WT genotype; Mix, metabolites expressed in both diet and genotype. *, indicates ambiguity or incomplete structural characterization.

### Exploratory network collection

Examination of the spectrum of metabolites represented in the top 20 exploratory networks (Table 2) led to the following observations: a) the protein networks were identified via metabolites differentially expressed in both genotype and dietary interventions, highlighting a complex interplay between these factors (Table 3); b) palmitate is represented in the majority (16) of the networks, followed by myristate (15); margarate (13); caprate (10); linoleyl-arachidonoyl-glycerol (10); linoleyl-linoleoyl-glycerol (10); laurate (10); histidine (4); sphingomyelin (2); 3-hydroxybutyrate (2); methylpalmitate (1); myristoylcarnitine (1); cysteine (1); 2`-deoxycytidine (1); uridine (1); 2-aminobutyrate (1); 1-(1-enyl-palmitoyl)-2-oleoyl-GPC (1) and adenine (1).

**Table 3:** Metabolite Heatmap.

Metabolite	Heatmap_Cluster	Category
1-(1-enyl-palmitoyl)-2-oleoyl-GPC	Enriched_WT	Genotype
2-aminobutyrate	Enriched_CTL	Diet
2`-deoxycytidine	Enriched_WT	Genotype
3-hydroxybutyrate	Enriched_WT	Genotype
adenine	Enriched_Tg	Genotype
caprate	Enriched_HF	Diet
cysteine	Enriched_WT	Genotype
histidine	Enriched_Tg	Genotype
laurate	Enriched_WT	Genotype
linoleyl-arachidonoyl-glycerol	Enriched_CTL	Diet
linoleyl-linoleoyl-glycero	Enriched_CTL	Diet
margarate	Enriched_HF	Diet
methylpalmitate	Enriched_HF	Diet
myristate	Enriched_HF	Diet
myristoylcarnitine	Enriched_Tg	Genotype
palmitate	Enriched_HF	Diet
sphingomyelin	Enriched_Tg	Genotype
uridine	Enriched_WT	Genotype

Heatmap_Cluster, cluster of the sPLS-DA heatmap where the metabolite is located. Category, type of cluster category.

In addition to the metabolites described above (histidine, 2-aminobutyrate, cysteine and 3-hydroxybutyrate) that are relevant to AD pathology, the following metabolites deserve special attention:

- *Palmitate, methylpalmitate, margarate, and myristate:* they are all fatty acid derivatives. Specifically, esters of fatty acids or fatty acid salts. Altered levels of these metabolites can impact membrane integrity, neuronal health, neuroinflammation, oxidative stress, mitochondrial dysfunction, amyloid precursor protein processing, neuronal signaling and synaptic function, establishing a connection between diet, fatty acid metabolism and AD-related pathophysiological changes [55].
- *Caprate and laurate*: are both saturated fatty acids that are metabolized into ketone bodies, which can serve as an alternative energy source for the brain. In addition, they may exert anti-inflammatory effects and there is some evidence suggesting that they may preserve synaptic function and structure. Lauric acid has been shown to stabilize Aβ42 oligomers which may influence amyloid distribution in the fatty acid-rich brain environment [56].
- *Linoleyl-arachidonyl-glycerol (LAG) and Linoleyl-linoleyl-glycerol (LLG)*: are lipid compounds, specifically glycerol esters of polyunsaturated fatty acids. LAG is linked to lipid signaling and metabolism and is part of the endocannabinoid signaling system that is being investigated for its potential to reduce neuroinflammation, enhance neuroprotection, and improve cognitive function in AD [57]. LGL is a bioactive lipid molecule that has gained attention in recent research for its potential role in neuroprotection by enhancing membrane fluidity and cellular signaling and its implications in neurodegenerative diseases [58,59].
- *Myristoylcarnitine:* is a carnitine derivative with neuroprotective effect by potentially improving mitochondrial function, reducing oxidative stress, and supporting brain energy metabolism [60].
- *Sphingomyelin:* is composed of a sphingosine molecule, a fatty acid chain, and a phosphocholine group (a choline-linked phosphate group). The sphingomyelin pathway is activated downstream of oxidative stress induced by Aβ and TNF-α, leading to increased sphingomyelinase activity and ceramide levels in the brain [61].
- *2’-Deoxycytidine:* a nucleoside analog that is a building block of DNA and is involved in several biochemical processes. While it does not have a well-established or direct role in AD pathology, its involvement in DNA metabolism and cellular processes may make it a potential candidate for further study in relation to AD, especially in the context of neuroinflammation and DNA repair mechanisms [62].
- *Uridine*: nucleoside that plays a critical role in the synthesis of RNA and phospholipids, which are important for cell membranes and signaling processes. It has been studied in several contexts, including its potential therapeutic effects in neurological conditions such as AD [63].
- *Adenine:* a purine base that yields adenosine, a compound that integrates the adenosinergic system, considered a homeostatic mechanism that could affect memory loss in AD [64].

### Overview of biological patterns across core modules and their key metabolite-protein connections

**Fig 3:**
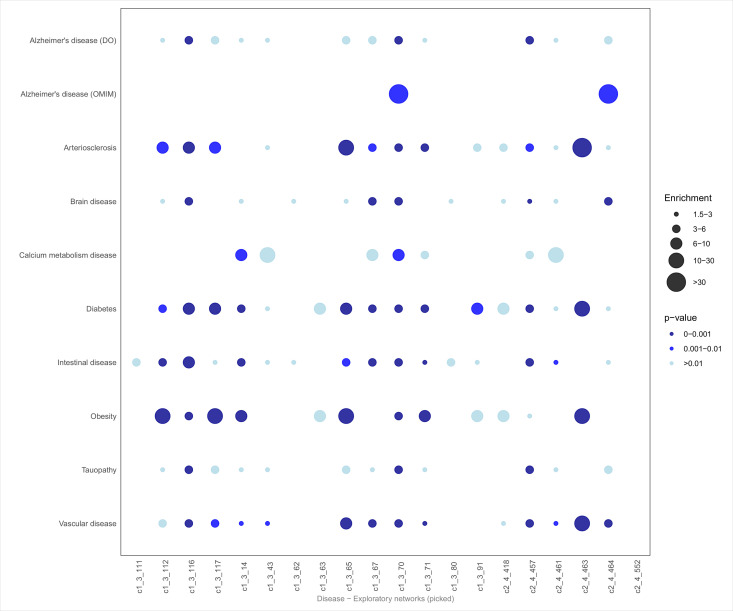
Disease enrichment dot plot. Bottom row, Top 20 exploratory Networks IDs. Left, significantly overrepresented Disease Ontology (DO) and manually collected OMIM categories related to AD. DO-OMIM class significance and enrichment score are calculated as the geometric mean of the p-values and mean of the enrichment scores, respectively, for all contained DO-OMIM categories.

**Fig 4:**
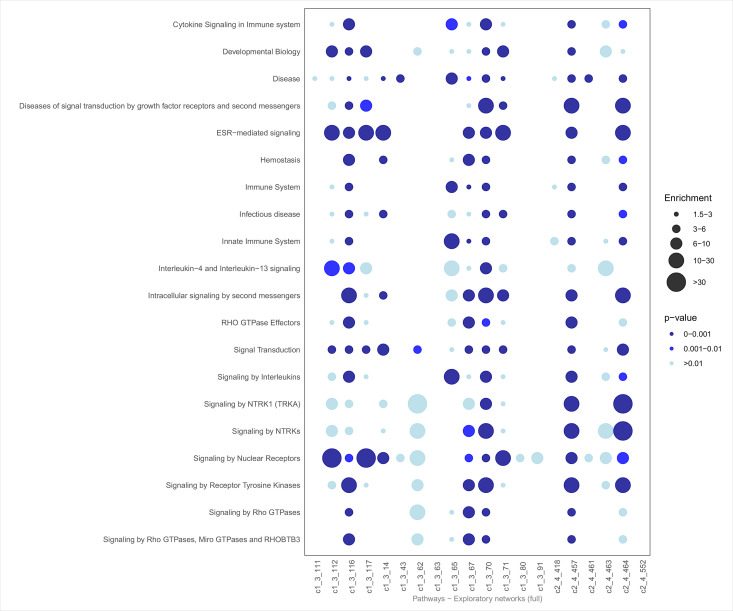
Pathway enrichment dot plot. Bottom row, Top 20 exploratory Networks IDs. Left, significantly overrepresented Reactome and WikiPathways categories related to AD manually collected. Reactome and WikiPathways class significance and enrichment score are calculated as the geometric mean of the p-values and mean of the enrichment scores, respectively, for all contained Reactome and WikiPathways categories.

**Fig 5:**
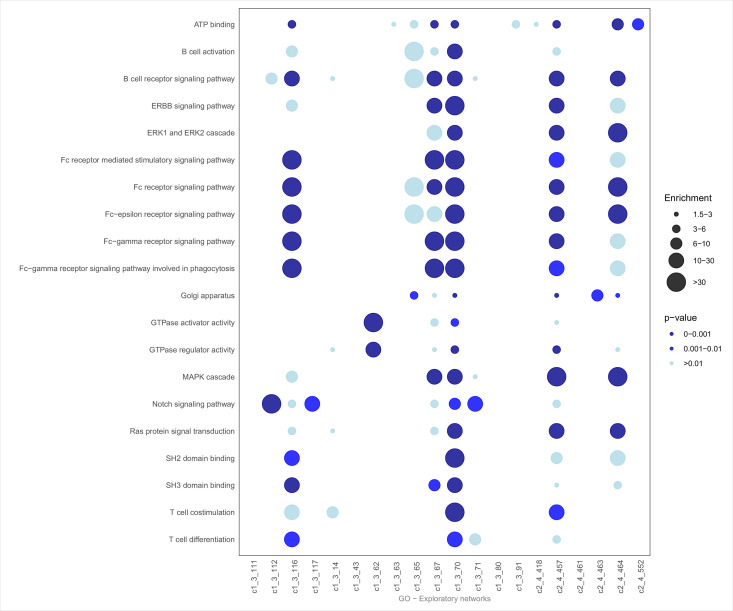
GO enrichment dot plot. Bottom row, Top 20 exploratory Networks IDs. Left, significantly overrepresented GO categories related to AD manually collected. GO class significance and enrichment score are calculated as the geometric mean of the p-values and mean of the enrichment scores, respectively, for all contained GO categories.

To investigate key biological patterns within the most significant exploratory networks, we explored the biology of the networks using over-representation analysis. For this, we used all biological annotations from ZS Revelen plus 19 additional custom AD-related gene sets. For a high-level view of all significant networks (Figs 3–5), we focused on three annotations: the enrichment in biological processes (imported from Gene Ontology), pathways (imported from Reactome and WikiPathways), and disease-associated proteins (imported from Disease Ontology and OMIM). Here, the average p-value was calculated (after p-value correction using the Benjamini-Hochberg procedure) across the 20 selected networks, retaining the top 20 annotations with the lowest average p-values. From this list, we prioritized annotations with *p*-values ≤ 0.01 and enrichment scores ≥ 1.5. Specifically, for Diseases, we also highlighted terms relevant to our research focus, such as AD, dementia, brain-related diseases, and conditions recognized as risk factors for AD.

The disease dot plot (Fig 3) revealed significant enrichment for terms associated with AD (both from OMIM and Disease Ontology), vascular disease, tauopathies, and brain disorders. Other enriched categories included diabetes, obesity, and atherosclerosis, which are known risk factors for neurodegenerative diseases. These findings underscore the relevance of metabolic and vascular processes in the studied networks.

The enrichment analysis for biological processes (Fig 4) highlights several immune and signal transduction pathways. Key annotations include T-cell differentiation and co-stimulation, MAPK cascade, and Fc receptor-mediated signaling. Binding activities, such as SH2 and SH3 domain binding, were also prominent, suggesting roles in intracellular signaling and immune responses.

Pathway analysis (Fig 5) showed notable enrichment for similar signaling mechanisms such as Rho GTPase signaling, receptor tyrosine kinase pathways, and MAPK family signaling cascades. Immune-related pathways, including interleukin signaling and cytokine signaling in the immune system, also featured prominently. Other significant pathways involved platelet activation and signaling, estrogen receptor (ESR)-mediated signaling, and processes linked to hemostasis and growth factor receptor signal transduction.

Out of the 20 exploratory networks, 12 showed a varying degree of enrichment in AD annotations. In general, networks enriched in AD-related proteins also displayed significant enrichment in diseases commonly recognized as risk factors for neurodegeneration, including obesity, atherosclerosis, and diabetes. Notably, networks c1_3_116, c1_3_70, and c2_4_457 showed the strongest associations with AD and tauopathy-related terms (Fig 3), while network, c2_4_464, stood out for its specificity, showing strong enrichment only in terms related to AD, brain diseases, and vascular conditions. This suggests that c2_4_464 network, which is modulated by diet, may be more closely linked to specific neurodegenerative and vascular processes compared to other networks. In addition to AD-related terms, 16 of the 20 networks showed enrichment in the “intestinal disease” category from the Disease Ontology. A smaller subset of networks (c1_3_14, c1_3_43, c1_3_67, c1_3_70 and c2_461) displayed enrichments for diseases related to calcium metabolism, highlighting the potential link between metabolic disturbances and neurodegenerative conditions.

When examining Gene Ontology (GO) overrepresentation, networks c1_3_116 and c1_3_70 again emerged as the primary drivers of significant enrichments, appearing in the majority of the top 20 GO terms (Fig 5). Key overrepresented GO terms included immune-related processes such as B and T cell activation, which were prevalent in over 50% of the networks. These terms, along with intracellular signaling processes, suggest that immune modulation and signaling cascades may play an important role in the biological context of these networks. Furthermore, a select group of networks (c1_3_67, c1_3_70, c2_4_457 and c2_4_464) showed enrichment in canonical proliferation-related pathways, such as ERBB2 and ERK signaling, which are known to be involved in cell cycle regulation and oncogenesis. Another subgroup showed significant enrichment for proteins expressed in the Golgi apparatus, implicating cellular trafficking and protein processing mechanisms in these networks.

In the Reactome and WikiPathways databases, significant pathway enrichment was observed in half of the networks, with many showing robust enrichment for the top 20 pathways (Fig 4). Immune-related signaling pathways emerged as particularly prominent, with interleukin-4 (IL-4) and interleukin-13 (IL-13) signaling being significantly enriched in several networks. These cytokines are known to influence immune responses and tissue repair processes [65,66], which may be relevant to neuroinflammation and neurodegenerative diseases.

Additionally, pathways associated with neurotrophic receptors (NTRK) and estrogen receptor (ESR)-mediated signaling were notably enriched. Both are involved in neuronal survival, differentiation, and response to estrogen, which has been implicated in brain-related diseases, including AD. Collectively, these findings highlight the central role of immune signaling, and neurotrophic factors in the biological networks under investigation.

### Relevant key modules detected in the set of exploratory networks and their association with Alzheimer´s disease neuropathology

**Fig 6:**
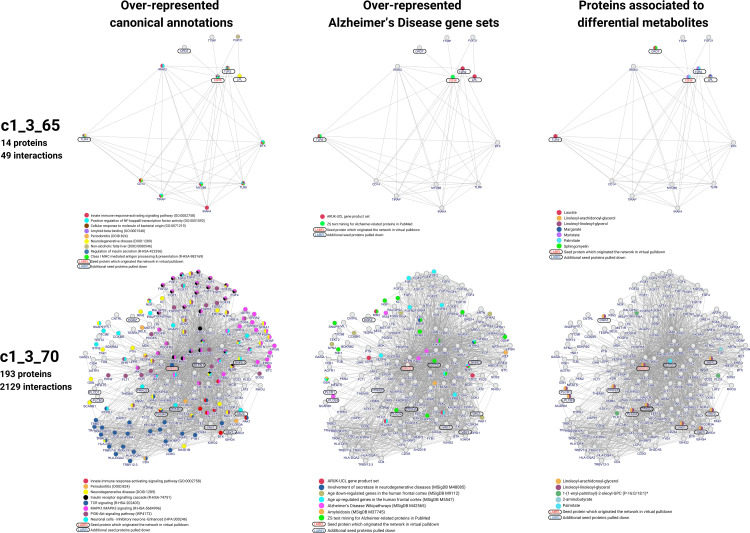
Structural features of the C1_3_65 and C1_3_70 networks. Circle, gene/protein annotated. Line, gene/protein with bibliographic evidence of association. First column, gene/protein labeled with pathway association; Second column, gene/protein labeled with AD gene sets association; Third column, gene/protein labeled with differential metabolite association.

As outlined in “Prioritization of key network modules” (Materials and Methods) of the 20 exploratory networks (Table 2), we selected for further exploring, two networks (Fig 6) potentially indicating biological relevance based on different criteria. The first, network c1_3_65, was chosen because it was the top hit in seed protein enrichment analysis (q-value = 4.2E-4; number of participating metabolites = 7), reflecting a high participation of metabolites in our model. The second network, c1_3_70 (q-value = 1.26E-3; number of participating metabolites = 5), was identified through its significant involvement in immune, metabolic, and AD-related pathways, highlighting its importance in disease mechanisms.

The c1_3_65 network is an accurate representation of the major biological pathways linked to the immune system. The relevant proteins implicated in this association are CD36, lipoprotein lipase (LPL), Toll-like receptor 2 (TLR2) and Toll-like receptor 4 (TLR4). The relationship between LPL and CD36 plays a crucial role in fatty acid (FA) uptake and triglyceride (TG) clearance. LPL catalyzes the hydrolysis of TG, whereas CD36 facilitates cellular FA uptake [67]. CD36 deficiency in mice results in elevated plasma FA and TG levels, impairing LPL-mediated TG hydrolysis [68]. It is of note that TLR4 and CD36 are two proteins involved in inflammation related to atherosclerosis and AD [69]. CD36, a receptor for oxidized LDL (OxLDL), contributes to foam cell formation, a key step in atherosclerosis. CD36 can recognize OxLDL even after short periods of oxidation, suggesting a role in scavenging early-stage modified LDL. In animal models of AD, the interaction between Aβ and CD36 has been consistently associated with a pro-inflammatory response [69]. Interestingly, CD36 and TLR4 interact to trigger inflammation in response to OxLDL and Aβ. This interaction involves the formation of a TLR4-TLR6 heterodimer, regulated by CD36 signaling. This mechanism suggests a novel model of TLR heterodimerization triggered by coreceptor signaling [70,71]. The observed increase in several metabolites within this network in the feces of Tg-HFD rats, as evidenced by the heatmap, suggests a potential dysregulation in the oxidation of dietary fatty acids. This dysregulation may be linked to the activation of the inflammatory pathway described previously. As shown in Table 2, this network was integrated by proteins connected to metabolites coming from clusters associated with Diet and Genotype, indicating a possible interaction between these two factors in the context of the disease. The structural feature of this network is depicted in Fig 6 showing the 13 proteins related with CD36 (seed protein) and its annotations. The functional implications of the associated metabolites suggest that CD36 is a central component in orchestrating immunity and metabolic pathways associated with diet and genotype.

The c1_3_70 network has proteins significantly associated with AD and with other diseases that are known AD risk factors (Fig 3). It is also significantly enriched in pathways known to be related to AD. This would indicate that this is a fairly canonical protein network. PCLG1, a member of the phospholipase C family, is the main protein from which the rest of the connections are derived. It functions as a signal transducer, generating second messengers that regulate various cellular processes [72]. PLCG1 is activated by receptor tyrosine kinases and adhesion receptors, mediating signaling through its lipase activity and protein interactions [73]. While the protein does not have a direct association with any AD database, many linked first-order proteins do (Fig 3). PLCG1 plays a crucial role in brain function and it is implicated in various neurological disorders. Highly expressed in the brain, PLCG1 is involved in neuronal cell functions mediated by neurotrophins, synaptic transmission and brain development [73]. Abnormal expression and activation of PLCG1 have been associated with epilepsy, depression, Huntington’s disease, and AD [73,74]. In the c1_3_70 network we can observe three clusters of proteins associated with different pathways linked to PCLG1 (Fig 6). One group of proteins associated with TCR signaling, another with MAPK signaling and finally a cluster associated with insulin signaling and the PI3K-Akt pathway. This aligns with the importance of this protein network in canonical events of cellular immunity and the activation by phosphorylation of a wide range of cellular processes. In the context of the AD, it would be less meaningful to focus solely on the set of proteins without considering the impact that di-glycerols, a differential metabolite associated with several proteins in the network (Fig 6), may have on disease progression. PLCG1 hydrolyzes phosphatidylinositol 4,5-bisphosphate to generate DAG and inositol 1,4,5-trisphosphate, which are essential for the activation of protein kinase C and for increasing intracellular calcium levels [75]. Additionally, PLCG1 is implicated in the regulation of DAG levels at the Golgi, where it facilitates post-Golgi trafficking of proteins [76]. Although the relationship is complex, di-glycerols could be indicative of differential activity of this canonical protein and its interactions. From these results we could conclude that since this network is very generic and mostly influenced by diglycerols, these metabolites emerge as relevant in the gut-brain interaction and deserve to be studied in depth.

### Study caveats and limitations

While this study provides valuable insights, there are aspects that could be expanded upon in future work. The sPLS-DA analysis required complete datasets for each individual, resulting in the exclusion of many samples, and in particular the small sample size (n = 3) for the WT-HF limited the power to detect differential metabolites in this group. Furthermore, the suboptimal initial sequence quality in our 16S rRNA V3–V4 data necessitated stringent quality filtering to retain only high-confidence reads; however, this approach also led to the exclusion of a substantial number of sequences. Additionally, the intrinsic limitations of this technique restrict its ability to fully capture the complexity of microbial communities, particularly in terms of species-level resolution and the detection of rare taxa. Nevertheless, the number of retained ASVs was sufficient for characterizing community variations in our study. In future work, we advocate for the use of techniques with greater coverage to enhance resolution and accuracy. The findings of this study open the door to additional avenues for investigation. A more efficient parameter tuning process could be achieved with enhanced computational resources, such as dedicated server infrastructure. The network construction in this study was highly based on physical protein-protein interactions. While this approach ensured a focus on direct, well-established associations, future versions of the analysis could incorporate more indirect functional associations, such as those based on upstream/downstream regulatory connections between proteins. Including these additional functional connections could provide a more comprehensive view of the underlying biological networks and potentially lead to the discovery of new, previously undetected findings. Despite these limitations, the study successfully identified more than 20 metabolome- and microbiome-associated networks potentially related to amyloidosis and diet, providing valuable preliminary insights into the precise definition of novel pathways underlying the disease biology.

## Conclusion

By analyzing metabolic profiles from plasma and feces of WT and Tg rats and connecting these with protein network biology through metabolite-protein associations, we were able to identify protein pathways strongly correlated with cerebral amyloid pathology, diet and microbiota. Our differential metabolite-enriched modules encompass a wide range of biological functions known to be implicated in AD and potentially modulated by the gut microbiome. The detection of known AD-associated proteins within these core modules underscores their potential utility as a framework for uncovering novel disease mechanisms in AD and expands the potential correlation of our differential metabolites with disease.

Here we show that the most relevant protein networks are mainly associated with pathways linked to the immune system. Understanding the molecular basis of PPIs within the immune system continues to be a vital area of research, as it paves the way for developing innovative treatments for a variety of diseases, including cerebral amyloidosis. A better understanding of the complexity of these networks and the roles of proteins involved in gut-, diet- and brain-related processes is crucial for designing experiments to elucidate the impact of diet on brain function.

## Supporting information

S1 Fig PCA plots of metabolome (feces and plasma) and ASVs. In first row (up): three plots including all the samples in the pre-processing step. In the second row (down): three plots with outlier (rat-26) removed.
(PNG)

S2 Fig Classification error rates – choosing number of components. Choosing the number of components in PLS-DA testing the model with 10 × 3-fold CV function in the entire dataset. Classification error rates (overall and balanced) are represented on the y-axis with respect to the number of components on the x-axis for each prediction distance presented in PLS-DA.
(PDF)

S3 Fig MetOrigin metabolite traceability analysis. A. Venn diagram representing potential origin of metabolites. B. Histogram of differential metabolites.
(PNG)

S1 Table List of metabolites detected in plasma and feces by UHP-LC-MS/MS (Metabolon). Sample: Type of sample from which the metabolite was detected.
(XLSX)

S2 Table List of Bacteria genera detected in feces by 16s rDNA seq. (XLSX)

S3 Table sPLS-DA differential metabolites. (XLSX)

S4 Table sPLA-DA discriminant variables. Effect: Effect of de variable over the component in sPLS-DA model; Component: The component where the variable is part of in sPLS-DA model.
(XLSX)

S5 Table MetOrigin metabolites source. Link: Reference ID of metabolite-origin source of information.
(XLSX)

S6 Table sPLS-DA differential metabolites identified for protein pulldown. (XLSX)

S7 Table Protein-metabolite pulldown. (XLSX)

S1 Appendix Microbiota data: Metadata and taxonomic classification. (ZIP)

S2 Appendix Protein networks with complete overrepresentation analysis annotations. (XLSX)

S3 Appendix Main network features and network overlap quantification. (XLSX)

S4 Appendix Network collection in XGMML format. (ZIP)

S5 Appendix List of MSigDB gene sets utilized for overrepresentation analysis. (TXT)
