# Correction: New insights into the molecular biology of Alzheimer’s-like cerebral amyloidosis achieved through multi‐omics approaches

**DOI:** 10.1371/journal.pone.0336509

**Published:** 2025-11-10

**Authors:** Lorenzo Campanelli, Juan M. Sendoya, Scott Brody, Pablo Galeano, Sonia Do Carmo, A. Claudio Cuello, Eduardo M. Castaño, Andrés Gonzalés-Jimenez, Julia Verheul-Campos, Dina Medina-Vera, Fernando Rodríguez de Fonseca, Rasmus Wernersson, Laura Morelli

The ninth author’s last name is incorrect. The correct name is: Julia Verheul-Campos.
