# Supplementary figures and images for: New insights into the molecular biology of Alzheimer’s-like cerebral amyloidosis achieved through multi‐omics approaches
Source: PLoS One. 2025 Sep 3;20(9):e0330859. doi: 10.1371/journal.pone.0330859 (PMC12407441; doi:10.1371/journal.pone.0330859)

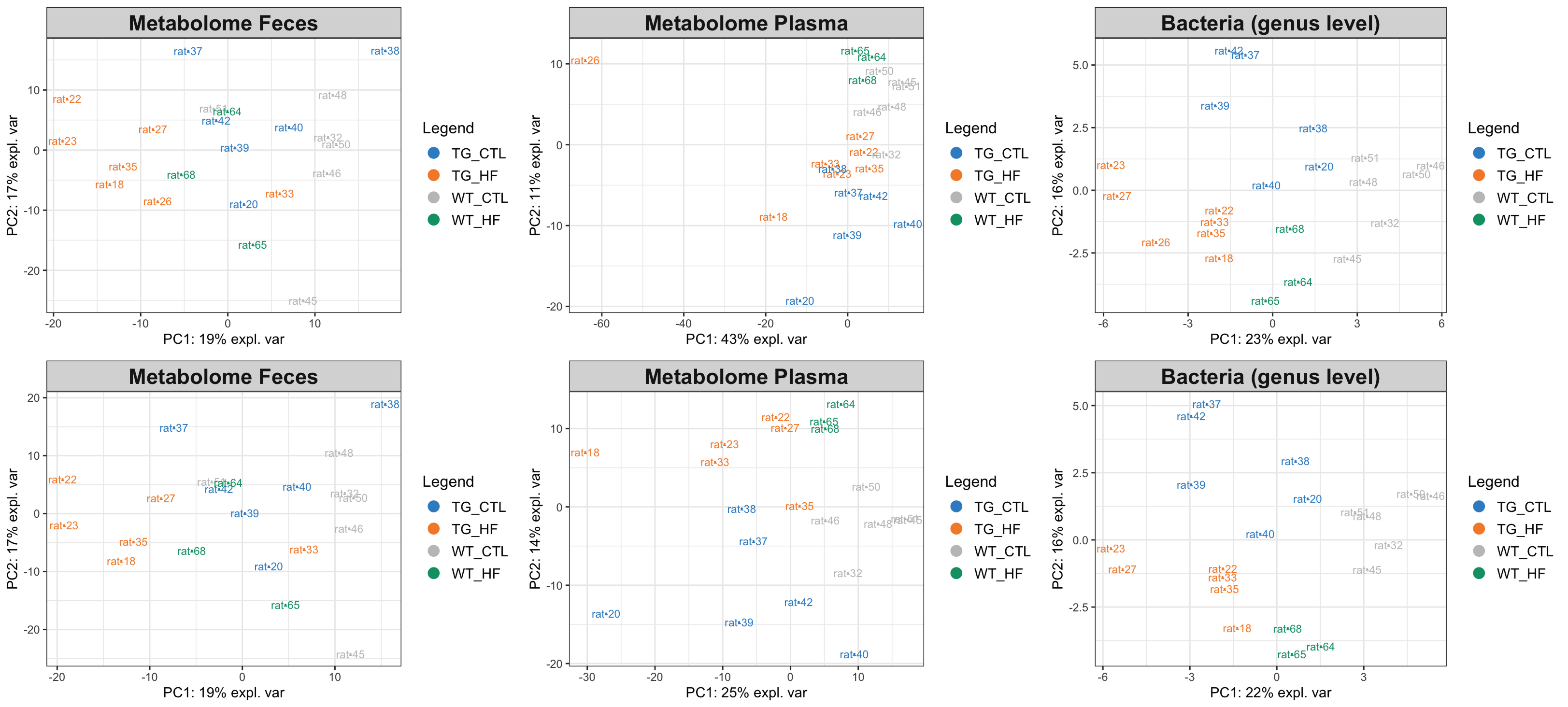

Supplement: S1 Fig — In first row (up): three plots including all the samples in the pre-processing step. In the second row (down): three plots with outlier (rat-26) removed. (PNG) [file pone.0330859.s001.png]

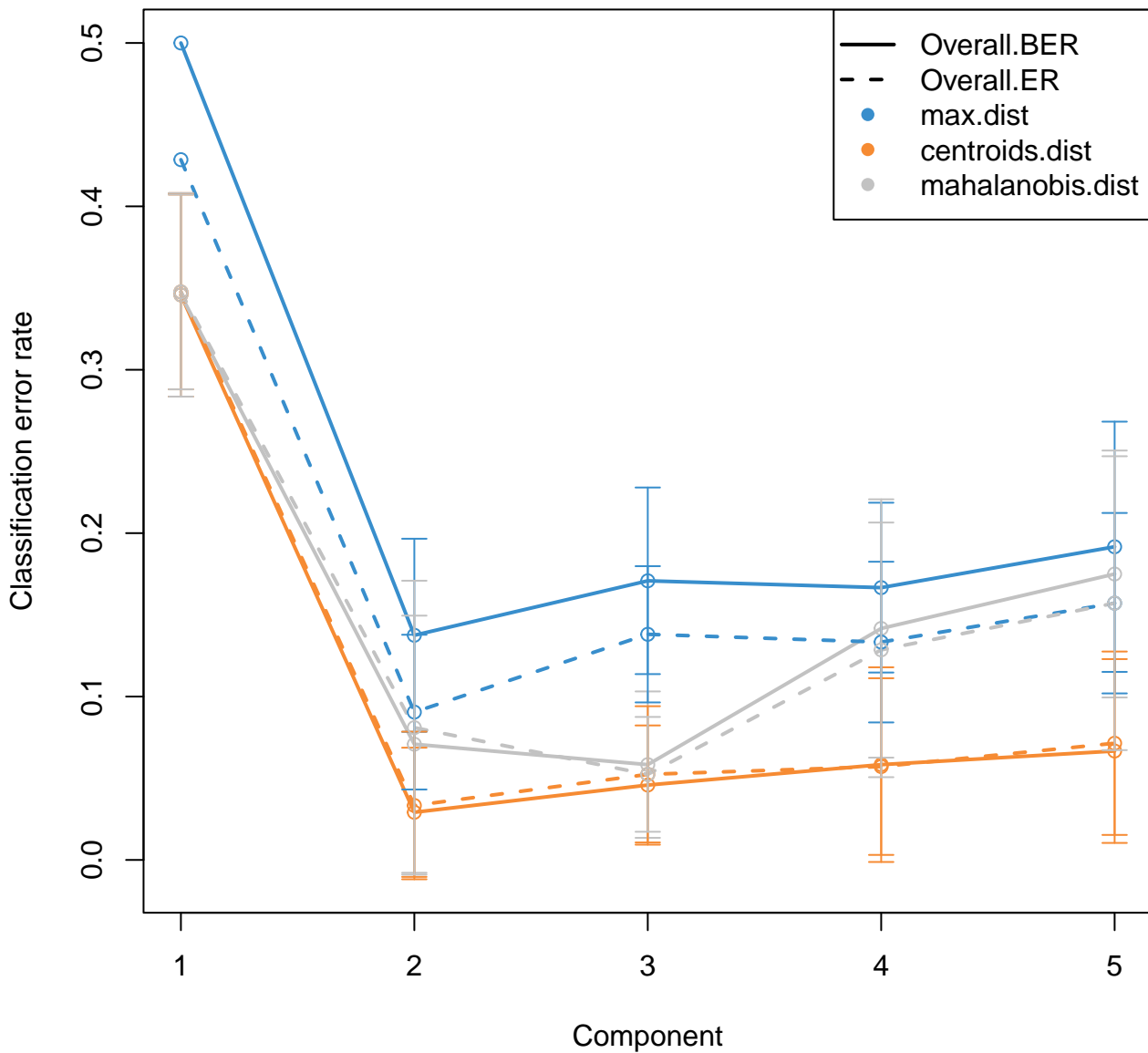

Supplement: S2 Fig — Choosing the number of components in PLS-DA testing the model with 10 × 3-fold CV function in the entire dataset. Classification error rates (overall and balanced) are represented on the y-axis with respect to the number of components on the x-axis for each prediction distance presented in PLS-DA. (PDF) [file pone.0330859.s002.pdf]

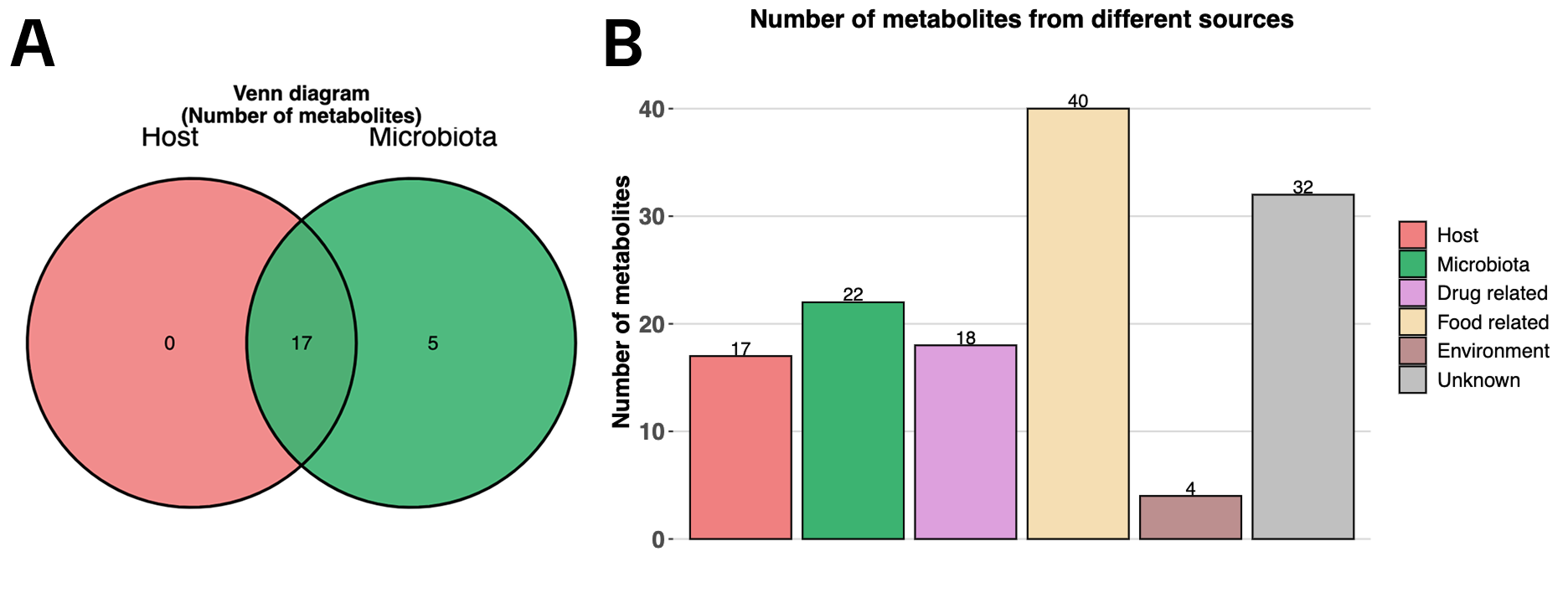

Supplement: S3 Fig — A. Venn diagram representing potential origin of metabolites. B. Histogram of differential metabolites. (PNG) [file pone.0330859.s003.png]
